# Editor's Note to ‘Autoregulatory circuit of human rpL3 expression requires hnRNP H1, NPM and KHSRP’

**DOI:** 10.1093/nar/gkac1099

**Published:** 2022-11-04

*Nucleic Acids Research*, Volume 39, Issue 17, 1 September 2011, Pages 7576–7585, https://doi.org/10.1093/nar/gkr461

The Editors were alerted in November 2021 that the anti-rpL7a blots depicted in Figure 1 show unusual levels of similarity.

The experiments were conducted over a decade ago, and the authors no longer have the original data. The authors have provided the following explanation; however, in the absence of original data, the Editors advise readers to examine the figure with care.

‘The panels were added at revision to satisfy a request from one of the referees who asked that an unrelated protein be tested. To respond to this request, the original immunoprecipitates with NPM, KHSRP and Ig G antibodies were loaded onto a new polyacrylamide gel and western blotting with anti-L7a antibodies was performed. To match the editing of Figure 1, the area of the autoradiography film corresponding to the molecular weight of L7a was selected (a restricted area corresponding to the four consecutively loaded samples that gave no signal) and this acquisition was divided and added as two different panels to Figure 1.

The strange similarity between the two panels may be because it is the area corresponding to the molecular weight of L7a of the same autoradiography film, rather than two different films as one might expect based on how Figure 1 was constructed.’

Julian E. Sale and Barry L. Stoddard

Senior Executive Editors

